# Construct Validity of the Brazilian Portuguese Version of the Thyroidectomy‐Related Voice and Symptom Questionnaire (BR‐PT‐TVSQ)

**DOI:** 10.1111/1460-6984.70183

**Published:** 2026-01-05

**Authors:** Ana Flávia de Sales Cândido, Jozemar Pereira dos Santos, Anna Alice Almeida, João Agnaldo do Nascimento, Leandro Pernambuco

**Affiliations:** ^1^ PhD Program in Decision and Health Models Federal University of Paraíba (UFPB) João Pessoa Brazil; ^2^ Department of Speech, Language and Hearing Sciences Federal University of Pernambuco (UFPE) Recife Brazil

**Keywords:** dysphonia, factor analysis, swallowing disorders, thyroidectomy, validation study

## Abstract

**Background:**

Impairment of voice and swallowing after thyroidectomy can negatively affect communication, eating and overall quality of life. The Thyroidectomy‐Related Voice and Symptom Questionnaire was developed to facilitate the early identification and monitoring of voice and swallowing outcomes in patients who have undergone thyroidectomy. While it has been translated and culturally adapted to Brazilian Portuguese, there is currently no evidence supporting its construct validity.

**Aim:**

To examine the construct validity of the Brazilian Portuguese version of the Thyroidectomy‐Related Voice and Symptom Questionnaire (BR‐PT‐TVSQ).

**Methods and Procedures:**

This cross‐sectional study included 395 Brazilian individuals undergoing thyroidectomy, mainly female (95.2%), with a mean age of 41.38 ± 11.12 years. Exploratory factor analysis (EFA) and confirmatory factor analysis (CFA) were used to test the structural validity of the 20‐item BR‐PT‐TVSQ. Internal consistency was assessed using Cronbach's alpha. Convergent and discriminant validity were analysed through internal correlations.

**Outcomes and Results:**

The EFA suggested a three‐factor model with a Cronbach's alpha of 0.947. The CFA confirmed the three‐factor model with acceptable goodness‐of‐fit indices: Factor 1 = voice symptoms; Factor 2 = oropharyngolaryngeal symptoms and Factor 3 = cervical and thoracic discomfort. All three factors presented significant convergent validity. The discriminatory power between Factors 2 and 3 was smaller than in other comparisons.

**Conclusions and Implications:**

The BR‐PT‐TVSQ provided evidence of construct validity for a three‐factor model with acceptable structure, convergent and discriminant validity. These sources of validity evidence are crucial to clinically ensure that the TVSQ‐PT‐BR structure accurately represents the investigated outcomes.

**WHAT THIS PAPER ADDS:**

*What is already known on this subject*

- The Thyroidectomy‐Related Voice and Symptom Questionnaire (TVSQ) was developed to identify and monitor symptoms related to voice, swallowing and neck discomfort in patients undergoing thyroidectomy. The TVSQ has already been translated and culturally adapted into Brazilian Portuguese (BR‐PT‐TVSQ). However, the BR‐PT‐TVSQ still lacks validation for other measurement properties, including construct validity.

*What this paper adds to existing knowledge*

- This study demonstrates that the BR‐PT‐TVSQ has construct validity, featuring three dimensions and acceptable levels of internal consistency, convergent validity and discriminant validity. These forms of validity evidence ensure that the structure of the instrument accurately represents the outcomes and dimensions being investigated, making it suitable for clinical use.

*What are the potential or actual clinical implications of this work?*

- The BR‐PT‐TVSQ is the only tool available in Brazilian Portuguese that has verified construct validity for the early assessment of symptoms related to voice, swallowing and neck discomfort in patients undergoing thyroidectomy. This questionnaire aids in decision‐making and the rehabilitation process.

## 1 Introduction

The thyroid gland is situated in the neck's median plane and near vital structures, such as the larynx, trachea, components of the carotid sheath, sympathetic chain, recurrent laryngeal nerve and mediastinal structures (Nam and Park 2017). Partial or total thyroidectomy may be indicated in some pathological conditions that can impact the function of the thyroid gland, including thyroiditis, multi‐nodular goitre and benign or malignant nodules (Nam and Park 2017).

The potential consequences of thyroidectomy include voice‐ and/or swallowing‐related issues, such as impaired voice pitch, voice fatigue, hoarseness, low loudness, a lump in the throat, dry throat and throat clearing (Araújo et al. 2017; dos Santos da Cruz et al. 2019; Iyomasa et al. 2019; Park et al. 2018; Pereira et al. 2003; Galluzzi and Garavello 2019). The prevalence of voice and swallowing symptoms following thyroidectomy ranges from 16% to 36% (Banach et al. 2013b). These symptoms may manifest during the preoperative period due to the compression (Araújo et al. 2017; dos Santos da Cruz et al. 2019; Iyomasa et al. 2019) and may persist or remit after surgery (Park et al. 2018; Pereira et al. 2003). They can have a significant negative impact on functionality and quality of life (Nam and Park 2017; Galluzzi and Garavello 2019).

The thyroidectomy‐related symptoms of vocal and swallowing disorders are multi‐factorial (Nam and Park 2017; Galluzzi and Garavello 2019). Such symptoms may result from temporary or permanent injuries to the recurrent laryngeal nerve or external branch of the superior laryngeal nerve, surgical extension, surgical technique, dissection of cervical muscles, orotracheal intubation, manipulation, stretching and fixation of the cervical muscles (Nam and Park 2017; Galluzzi and Garavello 2019; Davis et al. 2024). Even in the absence of postoperative complications, these symptoms may manifest as post‐thyroidectomy syndrome (Nam and Park 2017).

Screening for voice and swallowing disorders in patients undergoing thyroidectomy is essential (Davis et al. 2024; Chun et al. 2012; Chun et al. 2015; Hwang et al. 2022; Kim et al. 2020; Nam et al. 2012; Park et al. 2013). Instrumental assessments of laryngeal function before and after the procedure are considered best practices for these patients (Davis et al. 2024). However, access to these services may not be feasible for all patients within the Brazilian healthcare system. In Brazil, non‐validated patient‐reported outcome instruments have been used as a screening method to identify individuals with voice and/or swallowing symptoms (Araújo et al. 2017; dos Santos da Cruz et al. 2019). In Australia, patient‐reported outcomes are combined with flexible nasendoscopy in innovative service models for patients requiring vocal cord evaluations before and after thyroid surgery (Davis et al. 2024). However, these questionnaires were not specifically designed for this group of patients.

The Thyroidectomy‐Related Voice and Symptom Questionnaire (TVSQ) is a 20‐item screening questionnaire designed for patients undergoing thyroidectomy (Hwang et al. 2022; Nam et al. 2012). It is specifically intended to explore voice, swallowing and/or neck discomfort symptoms. The TVSQ has been employed by numerous studies as a screening instrument for the early identification of voice and/or swallowing symptoms, facilitating clinician decision‐making regarding referrals and monitoring patient progress (Chun et al. 2012; Chun et al. 2015; Hwang et al. 2022; Kim et al. 2020; Nam et al. 2012; Park et al. 2013).

To the best of our knowledge, the original TVSQ has already been translated and culturally adapted into Greek (Paspala et al. 2024), Arabic (Abaalkhail et al. 2023) and Brazilian Portuguese (BR‐PT‐TVSQ) (dos Santos et al. 2020). The content validity of the BR‐PT‐TVSQ was explored in a previous study (dos Santos et al. 2020). This translated and culturally adapted Brazilian Portuguese version of TVSQ was subsequently used in a Brazilian national survey involving 252 women who had undergone a thyroidectomy (de Sales Cândido et al. 2021). Nevertheless, no prior study has verified the construct validity of the BR‐PT‐TVSQ, considering the structural, convergent and discriminant validity (de Vet et al. 2011).

Structural validity investigates the dimensionality of the measurement instrument, the relationship between items, between items and total score and between items and the instrument's aim (Pernambuco et al. 2017; de Vet et al. 2011). Convergent and discriminant validity is based on formulated hypotheses to test construct validity of the instrument (de Vet et al. 2011). Clinically, it is important to understand these sources of validity evidence to ensure that the instrument accurately represents the investigated outcomes and dimensions. Therefore, the aim of this study is to explore the structural, convergent and discriminant validity evidence of the BR‐PT‐TVSQ.

## 2 Methods

This cross‐sectional study was approved by the Human Research Ethics Committee of the Health Sciences Center of the Federal University of Paraíba under the registration number 2.190.242. All participants signed an informed consent form. In addition, the study followed the guidelines of the *Standards for Educational and Psychological Testing* (SEPT) (AERA: American Educational Research Association, APA: American Psychological Association 2014, NCME: National Council on Measurement in Education), focusing on evidence of validity based on internal consistency.

The sample size was calculated by recommending at least 10 individuals per item for the factorial analysis (Nunnally 1978). Accordingly, it was anticipated that a minimum of 200 participants would be required. The study population included individuals living in all regions of Brazil who had undergone partial or total thyroidectomy, irrespective of the interval between surgery and data collection, and of any gender aged 18 years or above. Individuals who reported other surgeries in the head and neck area or those who could not answer the questionnaires or did not complete the BR‐PT‐TVSQ were excluded from the study.

Based on the criteria, the final sample consisted of 395 individuals. The majority were female (95.2%), with a mean age of 41.38 ± 11.12 years, a higher level of education and living in the Brazilian Southeast. They had undergone total thyroidectomy for a period exceeding 3 years, with or without radioiodine therapy or adjuvant radiotherapy (Table 1).

**TABLE 1:** Distribution of individuals undergoing thyroidectomy according to demographic and thyroidectomy‐related variables.

Variable	Category	*n* (%)
Biological sex	Female	376 (95.2)
	Male	19 (4.8)
Education background	Higher education	174 (44.1)
	Technical education	28 (7.1)
	Primary education	117 (29.6)
	No education declared	50 (12.7)
	Not informed	26 (6.6)
Region of residence	Southeast	190 (48.1)
	Northeast	122 (30.9)
	South	50 (12.7)
	Center‐West	24 (6.1)
	North	9 (2.3)
Time from surgery to data collection	Six months	19 (4.8)
	Between six months and a year	41 (10.4)
	Between one and three years	115 (29.1)
	Over three years	194 (49.1)
	Not informed	26 (6.6)
Type of surgery	Total thyroidectomy	371 (93.9)
	Partial thyroidectomy	24 (6.1)
Radioiodine therapy or adjuvant radiotherapy	Yes	192 (48.6)
	No	175 (44.3)
	Not informed	28 (7.1)

The data collection was based on the following three primary sources: (1) a query in the database of patients who responded to the BR‐PT‐TVSQ at the Head and Neck Cancer service of the Lauro Wanderley University Hospital (HULW/UFPB/EBSERH); (2) a telephone survey with patients who had received treatment at that same service but who had not responded the BR‐PT‐TVSQ in person and (3) those who completed the Google Forms web survey, which was accessible for 5 weeks via social and digital media, and which was designed to reach people from all five Brazilian geographic regions.

In both the original TVSQ (Hwang et al. 2022; Nam et al. 2012) and the BR‐PT‐TVSQ (dos Santos et al. 2020), the answer key for each of the 20 questions is on a Likert‐type scale from 0 to 4, according to the frequency of the symptom (never, almost never, sometimes, almost always and always). The total score is calculated by summing all items, with a range of 0–80, indicating a worsening of symptoms.

## 3 Exploratory Factorial Analysis (EFA)

To obtain the factorial model for explaining the correlation between items, an exploratory factorial analysis (EFA) was performed using the SPSS software, version 25, with the VARIMAX orthogonal rotation method employed as the factor extraction method.

The feasibility of EFA was verified through the performance of Bartlett's sphericity test and the Kaiser–Meyer–Olkin (KMO) measure (Whittaker and Schumacker 2022). Bartlett's sphericity test serves to accept or reject the null hypothesis that the correlation matrix is indeed an identity matrix. This indicates whether there are sufficient correlations to proceed with the analysis, with a significance level of less than 0.05. The KMO reference values range between 0 and 1 and are classified as excellent (0.90–1), good (0.80–0.89), median (0.70–0.79), mediocre (0.60–0.69), poor (0.50–0.59) and inadequate (0–0.49). Additionally, the sample adequacy measure (SAM) was employed, with values exceeding 0.50 retained for item inclusion in the subsequent analysis.

The principal components analysis method was employed for factor extraction, and the following criteria were utilized: (1) Kaiser's criterion: This evaluates the number of factors to be retained, leaving only factors with eigenvalues greater than 1. (2) Cattel's criterion: This graphically analyses the relationship between eigenvalues and the number of factors, with the point on the graph that becomes more horizontal indicating the maximum number of factors to be extracted.

The factor loadings and commonalities distributed across each factor were deemed acceptable, with values exceeding 0.5. The item‐total correlation and reliability analysis were conducted to ascertain the extent to which the items influenced the overall Cronbach alpha coefficient. The general Cronbach's alpha and the Cronbach's alpha of each factor with a value greater than 0.70 were acceptable.

## 4 Confirmatory Factor Analysis (CFA)

A confirmatory factor analysis (CFA) was conducted to assess the quality of fit of the factorial model provided by the EFA. The structural model was assembled using the AMOS Graphics module of the AMOS software. The maximum likelihood estimation procedure was employed to estimate the model parameters.

The following goodness‐of‐fit indexes (GFIs) and their threshold values were employed (Nunnally 1978; Whittaker and Schumacker 2022): chi‐square test >0.05; normed chi‐square <3.0, goodness‐of‐fit index (GFI), adjusted goodness‐of‐fit index (AGFI) and Tucker–Lewis index (TLI). Additionally, the comparative fit index (CFI) and the root mean square error of approximation (RMSEA) must both be greater than 0.90, while the parsimony goodness‐of‐fit index (PGFI) must fall between 0.60 and 0.80.

## 5 Convergent and Discriminant Validity

Convergent and discriminant validity verified the representation of the theoretical latent construct by the items of the BR‐PT‐TVSQ. Convergent validity is demonstrated when items are positively correlated, whereas discriminant validity is defined as the degree to which one factor construct is distinct from the others (Whittaker and Schumacker 2022; Hair et al. 2009).

To estimate the relative amount of convergent validity, we considered the factor loadings, mean extracted variance (MEV) and construct reliability (CR). All factor loadings must be statistically significant, with a minimum of 0.5 and an optimal value of 0.7 (Whittaker and Schumacker 2022). The discriminant validity was assessed by comparing the MEV of each factor with the square of the Pearson correlation between these factors (Whittaker and Schumacker 2022).

## 6 Results

### 6.1 Exploratory Factor Analysis

The sample was adequate for EFA according to the following criteria: KMO was 0.942, classified as excellent; Bartlett's sphericity test had a significance lower than 0.05 and the SAM of all items had a value higher than 0.5.

According to Kaiser's criterion, the factorial structure resulted in three factors with an eigenvalue >1. These three factors were named ‘voice symptoms’ (F1), ‘oropharyngolaryngeal symptoms’ (F2), and ‘cervical and thoracic discomfort symptoms’ (F3). The total variance of the three factors together showed an accumulated percentage of 67.69%. F1 had an eigenvalue of 10.061 and explained 50.303% of the total variance. F2 and F3 had eigenvalues of 2.054 and 1.424 and explained 10.269% and 7.122% of the variance, respectively. After a graphical analysis of the relationship between the eigenvalues and the number of factors present in the scree plot, the existence of three factors was confirmed by the Cattel method. All items had factor loadings greater than 0.5, and approximately 90.0% of the original variables had commonalities up to 0.60 (Table 2).

**TABLE 2:** Final results of the exploratory factor analysis of the BR‐PT‐TVSQ.

		Factor loadings			
		F1	F2	F3	
Question BR‐PT‐TVSQ (dos Santos et al. 2020)		voice symptoms	oropharyngolaryngeal symptoms	cervical and thoracic discomfort	Communality
Q8	*Sente dificuldade para falar alto/forte?* Do you find it difficult to speak loudly/strongly?	0.815			0.749
Q2	*Tem dificuldade para produzir sons agudos/finos?* *(entrevistado pode mostrar um exemplo de som agudo/fino)?* I have difficulty producing high pitch	0.807			0.715
Q1	*Tem dificuldade para cantar?* I have difficulty singing	0.805			0.704
Q9	*Sente que sua voz ficou fraca?* Do you feel your voice has become weak?	0.805			0.749
Q6	*Sente que sua voz está rouca e/ou com falhas?* I feel vocal fatigue after a long conversation	0.803			0.716
Q4	*Sente que faz esforço para falar?* I feel strained when producing voice	0.782			0.731
Q5	*Sente sua voz cansada depois de conversar por muito tempo?* I feel pain of discomfort after talking	0.777			0.716
Q3	*Sente que sua voz está mais grave/grossa?* I feel like my voice tone is lower than before	0.712			0.556
Q7	*Sente falta de ar quando fala?* Do you feel short of breath when you speak?	0.633			0.607
Q10	*Sente dor ou desconforto depois de conversar?* Do you feel pain or discomfort after talking?	0.605			0.617
Q13	*Precisa pigarrear frequentemente porque sente secreção em sua garganta?* Do you need to clear your throat often because you feel secretions in your throat?		0.866		0.792
Q11	*Sente muita secreção na garganta?* Do you feel a lot of secretion in your throat?		0.850		0.775
Q12	*Sente que tem algo preso na garganta?* Do you feel like there's something stuck in your throat?		0.689		0.667
Q14	*Tosse após comer ou deitar?* Do you cough after eating or going to bed?		0.670		0.658
Q15	*Percebe sua boca seca e sente sede?* Do you feel your mouth is dry and thirsty?		0.512		0.501
Q18	*Sente desconforto ou dor pela sensação de dormência nos ombros?* Do you feel discomfort or pain due to numbness in your shoulders?			0.823	0.731
Q16	*Sente desconforto ou dor pela sensação de dormência no pescoço?* Do you feel discomfort or pain due to numbness in your neck?			0.782	0.667
Q17	*Sente desconforto ou dor pela sensação de dormência no peito?* Do you feel discomfort or pain due to numbness in your chest?			0.759	0.661
Q19	*Sente desconforto quando come ou bebe?* Do you feel discomfort when eating or drinking?			0.593	0.609
Q20	*Sente dificuldade para respirar ou engasga com frequência?* Do you have difficulty breathing or choke frequently?			0.529	0.618
		Cronbach's alpha: 0.945	Cronbach's alpha: 0.869	Cronbach's alpha: 0.848	

The total Cronbach's alpha coefficient was 0.947, which is classified as excellent. The Cronbach alpha for each of the three factors exceeded 0.7. The instrument demonstrated consistency with the latent construct, indicating a high level of internal consistency (Table 2).

## 7 Confirmatory Factor Analysis (CFA)

**FIGURE 1:**
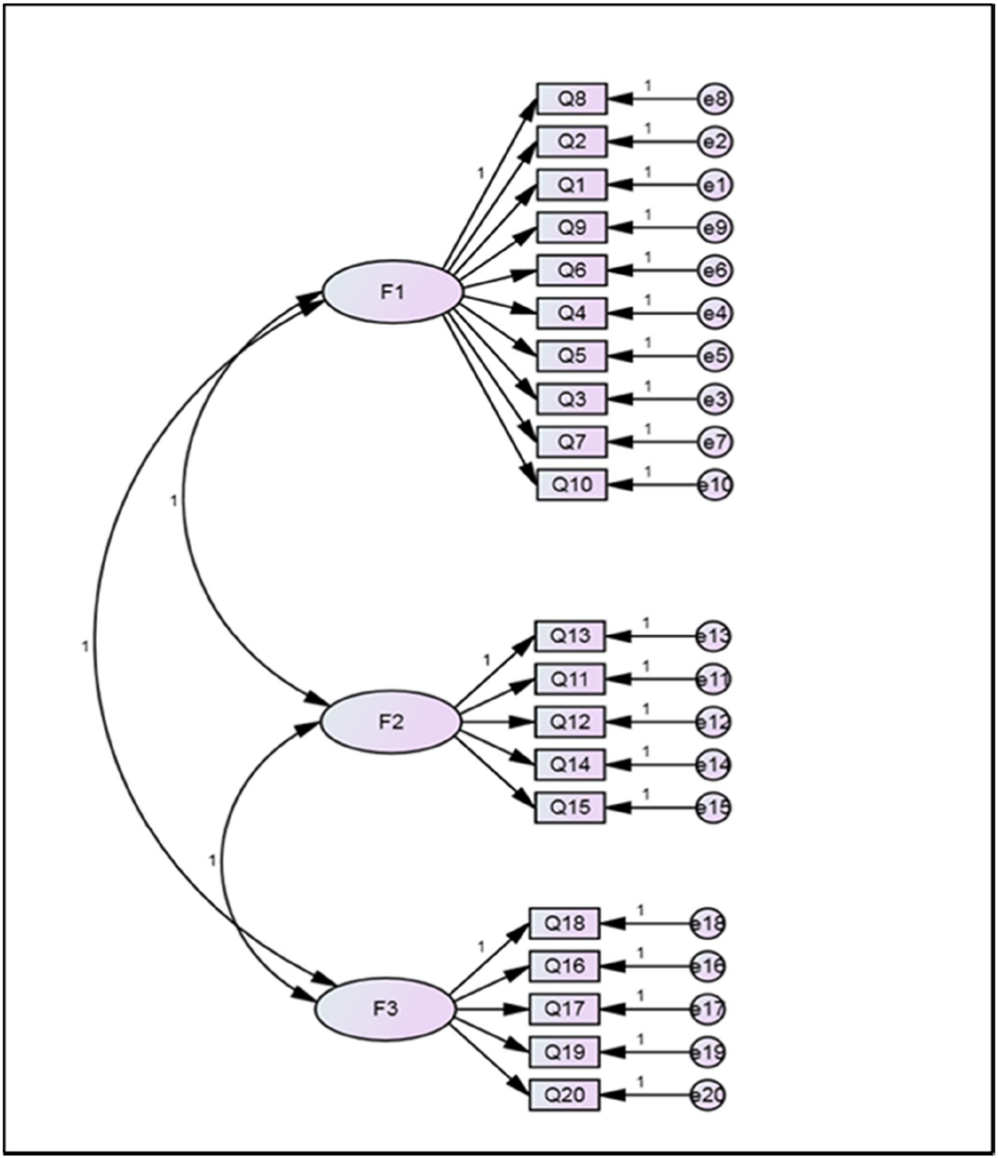
First CFA path diagram—three factors; 20 variables.

**FIGURE 2:**
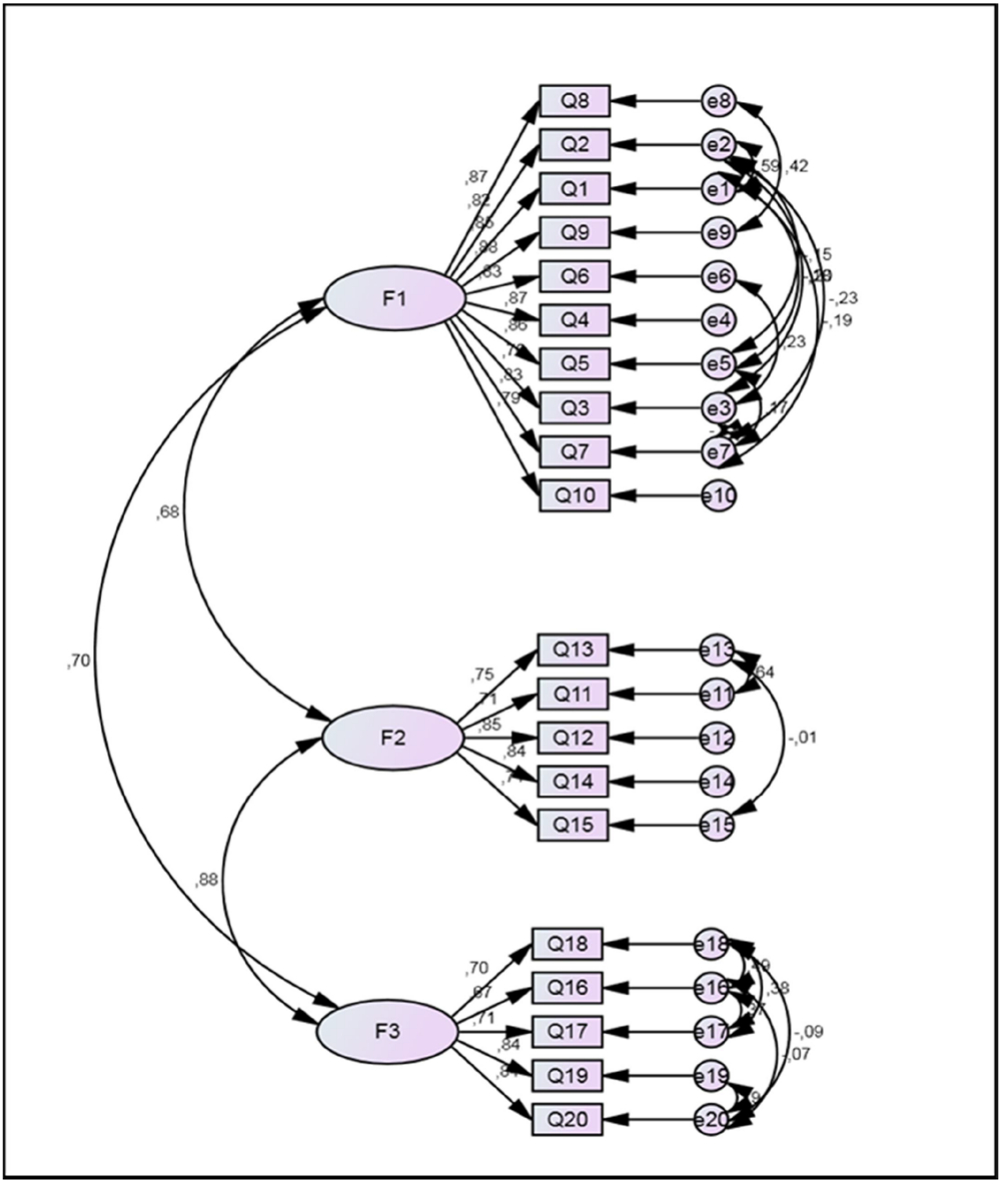
CFA path diagram of the standardized final model—three factors; 20 variables.

The CFA generated the measurement model through the path diagram (Figure 1), based on the structure provided by the EFA. However, the resulting model exhibited poor adjustment. A total of 39 outliers were excluded, and the correlation of errors proposed by the fit indexes of the initial model was incorporated. The path diagram of the second model, comprising 356 individuals, is presented in Figure 2.

The second proposed model demonstrated an acceptable level of goodness‐of‐fit. The GFI and RMSEA were above 0.9, and the AGFI exhibited a marginal value of 0.884. The relative fit indices TLI and CFI were greater than 0.9, and the parsimony fit index PGFI was greater than 0.6. The only test that did not meet the requisite fit criteria was the chi‐square test, with a *p* value of less than 0.05.

Additionally, a two‐factor CFA was conducted to assess the structure proposed by the original TVSQ, comprising the sub‐scales ‘voice changes’ and ‘throat and neck discomfort’. However, this model demonstrated inferior goodness‐of‐fit compared to the final three‐factor model (Table 3).

**TABLE 3:** Goodness‐of‐fit indices for the CFA models.

	Goodness‐of‐fit indices^a^							
CFA models	Chi‐square	Normed chi‐square	GFI	AGFI	PGFI	TLI	CFI	RMSEA
Two‐factor model (similar to the original TVSQ)	1 162 718^**^	6224	0.751	0.722	0.672	0.827	0.829	0.115
First three‐factor model (395 individuals)	884 592^**^	5234	0.805	0.757	0.648	0.859	0.874	0.104
Final three‐factor model (356 individuals)	336 483^**^	2214	0.916	0.884	0.663	0.963	0.970	0.058

^a^ Threshold values (Nunnally 1978; Whittaker and Schumacker 2022). chi‐square test > 0.05, normed chi‐square < 3.0, goodness‐of‐fit index (GFI), adjusted goodness‐of‐fit index (AGFI), Tucker–Lewis index (TLI) and comparative fit index (CFI) > 0.90, root mean square error of approximation (RMSEA) between 0.05 and 0.08 and parsimony of the goodness‐of‐fit index (PGFI) between 0.60 and 0.80.

** *p* < 0.05.

### 7.1 Construct Validity

All three factors showed a significant convergent validity, where all factor loadings have a value above 0.5; extracted variance has a value above 0.5; and CR has a value above 0.7 (Table 4).

**TABLE 4:** Statistics of the Factors 1–3 constructs or dimensions for the BR‐PT‐TVSQ instrument (*n* = 356).

Reliability and validity	Construct			Non‐standardized estimative	Standard error	C.R. (*t*)	Standardized estimative	*p* value
Reliability composite = 0.928	Q8	←	Factor 1	1	—	—	0.874	—
	Q2	←	Factor 1	0.903	0.043	21.020	0.819	^***^
	Q1	←	Factor 1	0.890	0.039	22.739	0.850	^***^
Extracted variance = 0.722	Q9	←	Factor 1	0.945	0.030	31.784	0.875	^***^
	Q6	←	Factor 1	0.910	0.042	21.843	0.829	^***^
	Q4	←	Factor 1	0.942	0.039	24.376	0.872	^***^
	Q5	←	Factor 1	0.992	0.042	23.435	0.862	^***^
	Q3	←	Factor 1	0.783	0.048	16.237	0.704	^***^
	Q7	←	Factor 1	0.889	0.041	21.445	0.828	^***^
	Q10	←	Factor 1	0.806	0.041	19.859	0.789	^***^
Reliability composite = 0.943	Q13	←	Factor 2	1	—	—	0.746	—
	Q11	←	Factor 2	0.893	0.040	22.515	0.708	^***^
	Q12	←	Factor 2	1.224	0.060	20.550	0.852	^***^
Extracted variance = 0.677	Q14	←	Factor 2	1.103	0.055	20.028	0.840	^***^
	Q15	←	Factor 2	1.410	0.068	15.388	0.713	^***^
Reliability composite = 0.957	Q18	←	Factor 3	1	—	—	0.699	—
	Q16	←	Factor 3	0.941	0.056	16.936	0.665	^***^
	Q17	←	Factor 3	0.858	0.050	17.271	0.707	^***^
Extracted variance = 0.669	Q19	←	Factor 3	1.014	0.053	19.215	0.840	^***^
	Q20	←	Factor 3	1.203	0.064	18.839	0.838	^***^

Table 5 presents that only Factor 3 × Factor 2 did not present discriminant validity since the extracted variance (arranged on the main diagonal) was lower than the shared variance. Thus, the discriminatory power between Factors 2 and 3 is smaller; thus, the two factors have very similar items.

**TABLE 5:** Comparison between the shared variance and the variance extracted from the measurement model of the Factors 1–3 constructs.

	Factor 1	Factor 2	Factor 3
	‘voice symptoms’	‘oropharyngolaryngeal symptoms’	‘cervical and thoracic discomfort’
Factor 1	0.722		
‘voice symptoms’			
Factor 2	0.462	0.677	
‘oropharyngolaryngeal symptoms’			
Factor 3	0.490	0.776	0.669
‘cervical and thoracic discomfort’			

## 8 Discussion

This study aimed to explore the construct validity of the BR‐PT‐TVSQ. The findings indicated a three‐factor model with acceptable structural, convergent and discriminant validity evidence.

The EFA resulted in the organization of the 20 items of the BR‐PT‐TVSQ into three factors, which was validated by the CFA. These three factors exhibited a total explained variance of 67.69%, exceeding the literature threshold (Whittaker and Schumacker 2022), indicating that the assembled factor structure is sufficient to explain the constructs and precludes the possibility of having residuals in the structure that could destabilize the sample (Damásio 2012).

The original TVSQ (Hwang et al. 2022; Nam et al. 2012) is divided into two sub‐categories, although this division was performed theoretically and not investigated by structural equation modelling, such as factorial analysis. The Arabic version of TVSQ (Abaalkhail et al. 2023) maintains the same sub‐categories from the original TVSQ and assesses only the internal consistency analysis. The Greek version of TVSQ (Paspala et al. 2024) employed both CFA and item response theory (IRT) analysis, testing models with two and one factor, respectively. The two‐factor model was determined to be superior in the Greek version.

Although a three‐factor model was fitted for the BR‐PT‐TVSQ, a two‐factor model was also tested. However, this latter model proved to be less satisfactory than the three‐factor model. The cultural adaptation of the TVSQ into Brazilian Portuguese resulted in operational equivalence, whereby the original items were transformed into interrogative sentences (dos Santos et al. 2020). It is possible that this may have an impact on the internal structure of the BR‐PT‐TVSQ in comparison to the other versions of the TVSQ. It is noteworthy that Factors 2 and 3 of the BR‐PT‐TVSQ lack discriminant validity, which warrants caution in interpreting the results.

The Factor 1 (‘voice symptoms’) was found to account for approximately 50% of the total variance of the BR‐PT‐TVSQ. Even after the surgical procedure was performed, the symptoms of voice changes were more prevalent than other symptoms. Among the potential consequences of thyroidectomy are damage to the laryngeal nerve, which, when affected, may result in paralysis or paresis of the vocal folds and alterations in the protection of the lower airways, leading to difficulty in producing high‐pitched sounds and difficulties in swallowing (Estrela et al. 2011). Patients who have undergone thyroidectomy are also likely to experience gastroesophageal reflux and changes in the mucosa of the vocal folds (Nam et al. 2012).

A number of studies have demonstrated that the symptoms of vocal disorders are the most prevalent in the population of individuals undergoing thyroidectomy (Banach et al. 2013a; Sahli et al. 2019). These symptoms are not always transient and may persist throughout an individual's life, affecting their quality of life (Kim et al. 2020; Cho et al. 2020; Choi et al. 2018; Martins et al. 2020). Furthermore, in the original study of the TVSQ (Hwang et al. 2022), patients who did not have sequelae, such as vocal fold paralysis, also exhibited voice complaints. The most common complaints were difficulties speaking loudly and producing high‐pitched sounds. This finding was replicated in the current study, whereby the items with the highest factor loading in Factor 1 were found to correspond precisely with the presence of these difficulties in voice.

Oropharyngolaryngeal symptoms were the second most frequently reported group of symptoms. These symptoms manifest as throat clearing after swallowing, effort to swallow, choking and a sensation of a lump in the throat (Araújo et al. 2017; dos Santos da Cruz et al. 2019; Martins et al. 2020). It is notable that these symptoms may persist even after treatment, a similar phenomenon compared to voice symptoms.

Factor 3 exhibited the lowest percentage of variance. This factor encompasses symptoms associated with discomfort in the shoulders, neck, chest and respiratory and swallowing difficulties. It was an expected finding because these were the least frequently symptoms reported by individuals who had undergone thyroidectomy. When they were reported, they manifested as numbness and limited movement of the neck and shoulders (Banach et al. 2013a).

This study presents some limitations. The sample was obtained through three different methods and still showed a predominance of females. However, besides ensuring a heterogeneous representation of the population studied across the country, this strategy also reflected a profile consistent with previous studies that used the instrument—from the original validation study (Nam et al. 2012), whose sample comprised 412 women and 88 men, to several subsequent investigations that also applied it (Chun et al. 2012; Chun et al. 2015; Kim et al., 2020; Park et al. 2013). It was not possible to conduct follow‐up assessments of the sample or to establish cause‐and‐effect relationships, although this was not the aim of the present study.

The main objective of this study was to enhance the validation process of a standardized, valid and reliable instrument in Brazilian Portuguese, focusing on evidence of validity based on internal consistency, thereby facilitating the management of thyroidectomy‐related symptoms by physicians. Nevertheless, the absence of other psychometric properties—such as external validity, test–retest reliability, responsiveness and measurement invariance—represents a limitation. These analyses are already being planned, and we expect to make the screening instrument increasingly robust for the target population. Despite these limitations, the study demonstrates consistency, with valid and representative results, culminating in an instrument that is widely used internationally and now validated for Brazilian Portuguese.

## 9 Conclusion

This study concluded that the BR‐PT‐TVSQ consists of three domains and demonstrates acceptable levels of internal consistency, convergent validity and discriminant validity. The validation process for the BR‐PT‐TVSQ should continue to investigate other measurement properties, including reliability and responsiveness.

## Funding

This study was financed in part by the Coordenação de Aperfeiçoamento de Pessoal de Nível Superior—Brasil (CAPES)—Finance Code 001.

## Ethics Statement

The study was approved by the Human Research Ethics Committee of the Health Sciences Center of the Federal University of Paraíba under the registration number 2.190.242.

## Consent

All participants in the study signed an informed consent form before data collection.

## Conflicts of Interest

The authors declare no conflicts of interest.

## Data Availability

The dataset cannot be shared for ethical reasons due to the rules of the institution's Human Research Ethics Committee.
